# Uncovering hidden insights in the chair rise performance of older adults using Dynamic Time Warping and K-means clustering

**DOI:** 10.1038/s41598-025-91015-x

**Published:** 2025-03-05

**Authors:** Ole Meyer, Rebecca Diekmann, Sandra Hellmers, Andreas Hein, Anna Schumacher

**Affiliations:** 1 Department of Health Services Research, Assistance Systems and Medical Device Technology, Carl von Ossietzky Universität Oldenburg, Ammerländer Heerstr. 114-118, Oldenburg, 26129 Germany; 2 Department of Health Services Research, Junior Research Group “Nutrition and Physical Function in Older Adults”, Carl von Ossietzky Universität Oldenburg, Ammerländer Heerstr. 114-118, Oldenburg, 26129 Germany

**Keywords:** Health care, Geriatrics, Public health

## Abstract

The five time chair rise test (5CRT) is commonly used in geriatric medicine and research to assess functional capacity and lower extremity strength to detect early age-related changes in older adults. Traditional stopwatch-based analyses may mask temporal variations in 5CRT transitions due to averaging. Temporal variations and dynamic characteristics are better assessed by motion variability analysis. This work employs k-means clustering using Dynamic Time Warping (DTW) as a metric for 5CRT to examine compensation mechanisms of older adults. The observational study included 172 healthy, community-dwelling adults aged 70+, yielding 860 chair rises recorded on a force plate and clustered using k-means. Descriptive statistics summarized performance distribution across clusters. Optimal clustering revealed two movement patterns, differing significantly (p $<0.01$) in 5CRT duration and forces during the stabilization phase. These patterns did not correlate directly with shorter or longer 5CRT durations, indicating overlap and highlighting the limitations of traditional stopwatch methods. This study demonstrates the potential of DTW and k-means clustering in geriatric medicine and research, enabling analysis of 5CRT performance independent of temporal variations, identifying potential health issues undetectable by conventional methods. The k-means model can be further trained to automate analysis, enhancing insights from 5CRT.

## Introduction

**Fig. 1:**
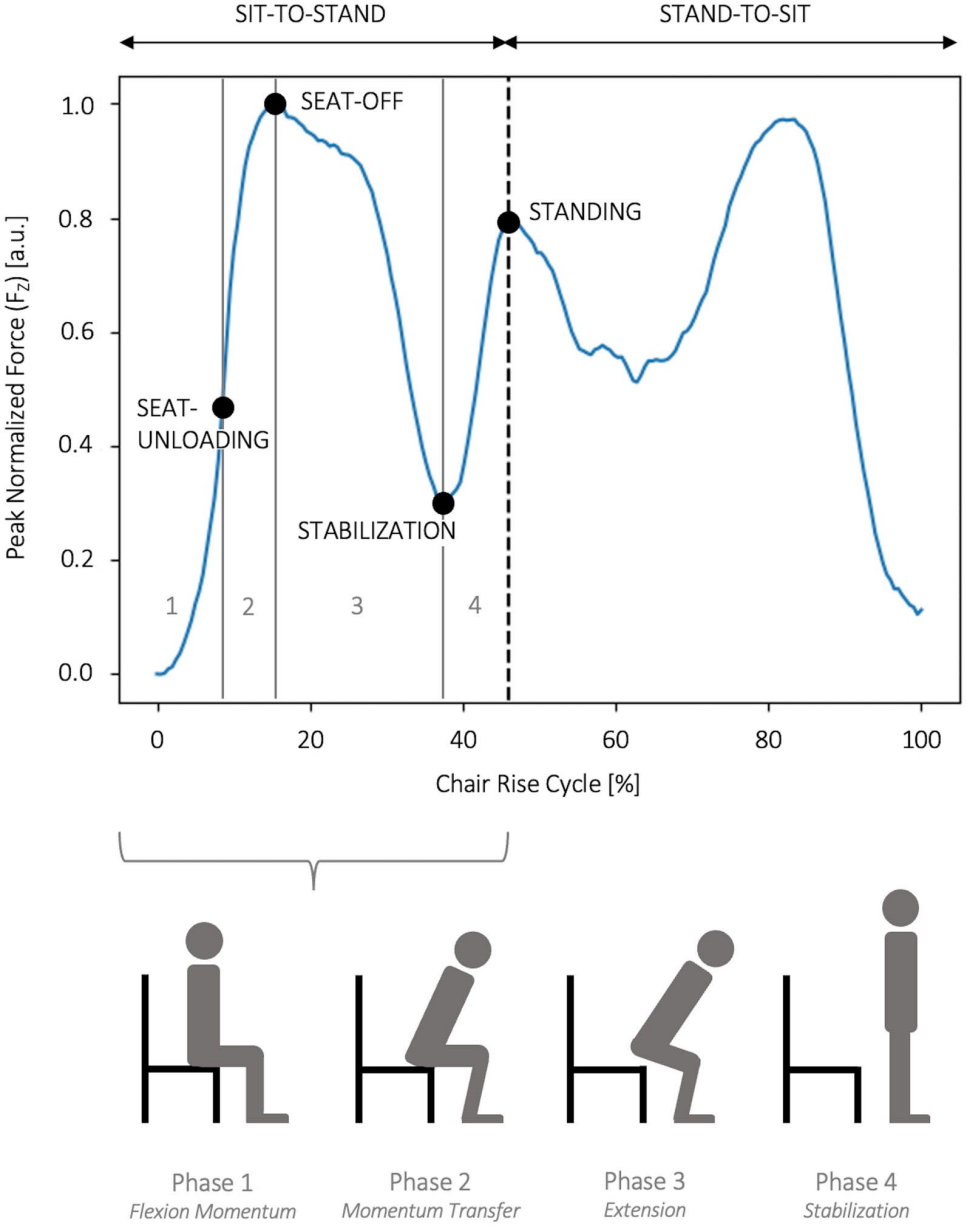
The different phases of the sit-to-stand part of the 5CRT according to Schenkman et al., 1990^16^ as seen in the $F_z$-component of the GRF data of a random participant. Phase 1-*Flexion Momentum*: Initiation of the movement. Phase 2-*Momentum Transfer*: Seat-unloading until maximum dorsiflexion is reached. Phase 3-*Extension*: The buttocks leave the chair and the whole body moves upward. Phase 4*-Stabilization*: The hip and knee joints are fully extended. When the participant stands fully upright, the sit-to-stand transition is completed.

Healthy aging and prolonging the independence of older adults is one of the major concerns of the world’s aging population^1,2^. According to the World Health Organization (WHO), healthy aging is the process of developing and maintaining the functional capacity that enables well-being in old age^2^, but also declines with age^3^. The five time chair rise test (5CRT) is a commonly used assessment in geriatric medicine and research to evaluate functional capacity and lower extremity strength, including balance and coordination to detect early age-related changes in older adults^4,5^. In this test, subjects are asked to sit in the center of an armless chair with their hands crossed over their chest. After a start command, they rise from a sitting position to a standing position (sit-to-stand) and vice versa (stand-to-sit) five times as fast as possible (see Fig. 1). The traditional approach is to measure the time needed to complete five chair rises using stopwatches as a single indicator^4,5^. Therefore, a shorter test duration indicates better performance. Although this may seem like a straightforward test, it does not take into account the nuances of how older adults perform the 5CRT.

The sit-to-stand and stand-to-sit tasks are complex and demanding motor activities that require adequate body mechanics to overcome body weight resistance. Large joint torques and precise balance control are essential for transferring body weight from a stable three-point based position in sitting to a more unstable two-point based position in standing^6–10^. A certain amount of variability is natural when a person moves and repeats a particular movement many times. While this variability in movement is natural, increased variability in repeated movements such as the sit-to-stand and stand-to-sit task is generally an indication of stabilization problems^11^. Since age-related mobility limitations do not occur immediately with the onset of physical decline, compensation is a common human movement strategy from the onset of physical decline until functional limitations occur^3^. Therefore, the nuances of how older adults perform the 5CRT, such as the duration of individual cycles and distinct transitions, can provide valuable insights into their physical abilities.

While traditional methods of 5CRT analysis, such as mean velocity and acceleration, may mask temporal variations in the transition due to averaging processes^11,12^, temporal variations and dynamic characteristics are better assessed by motion variability analysis. To uncover the nuances of older adults’ 5CRT performance, we evaluated the 5CRT cycles to gain insight into their movement mechanisms by clustering them according to patterns found in their applied force. A previous study of the cross-correlation between 20 participants of the same assessment concluded that Dynamic Time Warping (DTW) could prove to be a more robust method to investigate the variability in 5CRT performance^13^. Contrary to Euclidean distance estimates between time series, DTW offers greater flexibility and precision. Using DTW enables one to compare time series of different lengths without the need to interpolate one of the series. This interpolation can distort the shape of the investigated time series and cause similarities between the uninterpolated signals to ’disappear’. Therefore, DTW is used as it is a powerful time series analysis technique to efficiently measure the similarity between data sequences or time series of different time lengths by minimizing the effects of distortion and shift in the data. To build upon this foundation, machine learning methods such as K-means clustering are invaluable for the discovery of patterns within large and complex datasets. K-means clustering was selected for this study due to its efficiency, simplicity, and capacity to group data points with similar characteristics into meaningful clusters. The combination of DTW as the metric basis with K-means clustering allows for the analysis of temporal patterns and the classification of time series data in ways that conventional methods often fail to achieve. This powerful combination enables the identification of subtle temporal patterns, such as those present in sit-to-stand and stand-to-sit tasks, and allows for an investigation of these patterns from the perspective of the movement mechanisms employed by older adults performing 5CRT. The purpose of this work is to approach K-means clustering based on DTW analysis as a technique for sit-to-stand and stand-to-sit tasks from the perspective of the compensation mechanism of older adults performing 5CRT.

## Methods

### Study population

The study included a cohort of healthy, community-dwelling adults^14,15^. Recruitment occurred in various settings, including sports clubs, senior appointments, music societies, rehabilitation sports centers, physiotherapy departments, and through newspaper advertisements. Functional geriatric assessments were performed systematically, with strict inclusion criteria including the following parameters: attainment of a minimum age threshold of 70 years, community-dwelling, absence of major acute morbidities (e.g., pulmonary, renal, or cardiac), ability to climb ten flights of stairs without difficulty, self-sufficiency to attend assessment sessions, absence of pacemakers or other electronic implants, and successful completion of a timed “Up & Go” test within a time threshold of less than 20 s. After a telephone interview with each participant, a written informed consent form was provided at least 1 week before the assessment. Each participant then signed the informed consent form. In addition, a medical history was obtained, including a semi-structured questionnaire on health status (hypertension, history of stroke, chronic diseases such as diabetes mellitus and COPD, falls, and general health) and a review of medications. Blood pressure was measured for safety reasons. A value > 180/95 mm/Hg resulted in the participant’s exclusion from the study.

The research protocol has been reviewed and approved by the Medical Ethics Committee of the Hanover Medical School, Germany (No. 6948). The study was carried out in accordance with the approved guidelines and regulations.

### Experimental procedures

In this longitudinal observational study with two follow-ups, eligible participants were assessed at the study center at the Carl von Ossietzky Universität Oldenburg, Germany. A total of three assessments were performed: a baseline assessment (t0), an assessment after six months (t1), and an assessment after 24 months (t2). Before each assessment, each participant’s height and weight were measured using a stadiometer. After which, the participants completed the handgrip strength test, the stair climb power test, the timed “Up & Go” test, the short physical performance battery (SPPB), including the 5CRT, the 4-m gait speed test and balance test (semi-tandem stand, tandem stand), and the six-minute walking test. The tests were always performed in a standard manner. The primary focus of this work will be on the analysis of the 5CRT at the t2 assessment, as a height-adjustable chair was introduced to the setup starting from t2 onwards. By using the data gathered with the height-adjustable chair, the test is standardized and equitable, as it accounts for participants’ varying leg lengths and body proportions.

#### The five time chair rise test (5CRT)

Participants completed the 5CRT by rising from a chair to a stable standing position and sitting down again as fast as possible according to Guralnik et al.^4^. They were seated in the center of a height-adjustable chair with no armrests and, for consistency, started with their knees bent at a 90-degree angle and their feet placed shoulder-width apart on the floor. A goniometer was used to ensure the correct knee angle. Their arms were crossed in front of their chest. For the test, each participant had to stand up completely (sit-to-stand) and then sit down again (stand-to-sit), repeating this action a total of five times (see Fig. 1). Each test was performed without the use of arms for support. A command was given to start the test and the stopwatch was started to record the time taken to complete the test. The stopwatch was stopped once the participant had completed five chair rises and had touched the chair with their back. This measurement was conducted by a trained physiotherapist. Based on the time (in seconds) taken to perform the 5CRT, a score ranging from zero (which is the worst score) to four points (which is the best score) was assigned according to Guralnik et al.^4^ (see Table 1). This scoring method is usually used as part of the performance assessment of the SPPB and was therefore also used in this study. In addition, a force plate (AMTI AccuPower, Watertown, MA, USA) was used at a sampling rate of 200 Hz to measure the ground reaction forces (GRF) exerted on the ground during the task. The force plate was placed in front of the chair so that participants placed their feet upon it during the 5CRT. The plate was not moved during or between assessments. Additionally, the plate was calibrated and zeroed before each assessment. This involved entering the sex, height and weight of each participant into the provided software. Forces are presented in medial-lateral ($F_{x,GRF}$), anterior-posterior ($F_{y,GRF}$), and vertical ($F_{z,GRF}$) directions. A Microsoft Kinect V2 depth camera (30 fps) was also used to record the movements in three dimensions to correlate these movements with the force plate data for biomechanical signal interpretation.

**Table 1:** Scoring of the 5CRT based on measured test duration as used in the SPPB as a frailty measure according to Guralnik et al.^4^.

Score	5CRT duration
4 points	$\le 11.19$ s
3 points	11.20–13.69 s
2 points	13.70–16.69 s
1 points	16.70–60 s
0 points	$>60$ s or unable to complete the 5CRT

A higher score indicates a better performance.

When analyzing the sit-to-stand movement, dividing the movement into four distinct phases according to Schenkman et al., 1990^16^ provides valuable insight into the biomechanics and coordination involved (see Fig. 1):

Phase 1-*Flexion Momentum* begins with the initiation of the movement and extends to the point just before the buttocks leave the chair (Seat-Off). This initial phase includes flexion of the trunk and pelvis. There are also observable anticipatory actions that precede the visible beginning of movement. In particular, the head-arms-trunk segments are the primary contributors to the propulsion of the body forward before Seat-Off. Phase 2-*Momentum Transfer* begins when the buttocks lift off the seat and continues until maximum ankle dorsiflexion is reached. During this phase, the body’s center of mass (COM) shifts noticeably as it moves forward and upward. Phase 3-*Extension* follows immediately after maximum ankle dorsiflexion is achieved and when the buttocks left the chair (Seat-Off). Phase 3 concludes with full extension of the hip and knee joints. Phase 4-*Stabilization* marks the successful completion of the sit-to-stand transition with the participant standing upright.

### Data analysis

Data analysis was performed using custom functions in Python^17^, including the *tslearn* package^18^ to apply various analysis methods. These included DTW, which was used to estimate the similarity between the chair rises performed by a single participant, as well as the similarity of chair rises performed by multiple participants. This metric was also used in other methods such as k-means clustering, which was also provided by *tslearn*^18^. For DTW and clustering the peak-normalized vertical component ($F_{z,GRF}$) of the recorded GRF data was used. For quantification purposes, the vertical component ($F_{z,GRF}$) was normalized by dividing the participants body weight ($F_{z}=F_{z,GRF}/mg$) according to Hof^19^.

#### Quantification of 5CRT duration

The first step in the data analysis was to visually inspect the camera system recordings to check the participants’ postures and movements and associate them with the force plate data for a first impression. The force plate data were then labeled by manually determining the start and end within the recorded vertical force data of each of the five chair rises performed by each participant. This was done by finding the point at which the force plate was at rest before and after the 5CRT. In order to examine the relationship between the duration of the 5CRT measured by the stopwatch and that obtained from the force plate measurement of the participants, a comparison will be made between both of them.

#### Dynamic Time Warping (DTW)

DTW determines the similarity between time series of different lengths by finding the optimal alignment between the compared time series in the form of a ’warping path’. For this, every point of each time series is mapped to a point on the other, while the Euclidean distance between the points is minimized. Therefore, the resulting similarity score is determined by an optimization problem that seeks to find the optimal transfer function for which the Euclidean distance between the two time series is minimal. The process of DTW is defined as follows^18^:

Given two time-series $x = \left[ x_1,x_2,x_3,...x_M\right]$ and $y = \left[ y_1,y_2,y_3,...y_N\right]$ of length *M* and *N*, a *MxN* cross-similarity matrix is determined containing the Euclidean distance $d(x_i,y_j)$ between every data point contained within *x* and *y*. The optimal path $\pi = \left[ \pi _1,\pi _2,\pi _3,...\pi _K\right]$ minimizing the sum of the Euclidean distances $d(x_i,y_j)$ between the points $\pi _1 = (0,0)$ and $\pi _K = (M,N)$ is then called DTW path. This path determines the similarity score between the time-series and is defined by the following optimization problem:

$$\begin{aligned} DTW (x,y) = min_\pi \sqrt{\sum _{(i,j) \epsilon \pi }d(x_i,y_j)} \end{aligned}$$

DTW can also be used to determine the Barycenter $\mu$ of a set of time series *D*^18^. This Barycenter is a new time series with the best possible DTW similarity score to every time series within the set *D* and is determined by the following optimization problem:

$$\begin{aligned} min_\mu \sum _{x \epsilon D} DTW(\mu ,x) \end{aligned}$$

In this work, a variation of DTW called soft-DTW according to Cuturi & Blondel^20^ is applied to visualize the Barycenters of time series sets. In contrast to DTW, soft-DTW does not use the non-differentiable ’min’ operator and thus achieves a more smoothed DTW path and a Barycenter that closely matches the time series within the set, as presented in Cuturi & Blondel^20^.

#### K-means clustering

K-means clustering is an unsupervised machine learning algorithm used to divide a dataset into distinct groups, or clusters, based on similarity patterns in the data. In this work, clustering was based on the DTW Barycenter Averaging (DBA) algorithm as presented in Petitjean et al.^21^, which allows the k-means clustering methodology to be applied to time series data. This algorithm is based on the idea of clustering a set of time series based on iterative improvement upon an initial Barycenter. Which ultimately results in multiple Barycenters splitting the dataset into clusters based on the individual time-series DTW similarity to these Barycenters. As k-means algorithms tend to converge to local minima, it was run 10 times with differing centroid seeds to ensure the best output. Another parameter of the k-means are the maximum iterations which was set to 100.

#### Quantification of 5CRT clusters

To quantify the different clusters, the force applied during different phases^16^ of the 5CRT is investigated within this work. This is done by normalizing the force measured by the force plate by dividing it by each participant’s bodyweight ($F_z = F_{z,GRF}/ mg$) according to Hof^19^. Increased focus is applied to the sit-to-stand portion of the 5CRT, with the vertical force during Seat-Off and applied during the first stabilization phase. These values should provide information about participants’ performance and indicate participants’ stabilization challenges.

### Evaluation

#### K-means evaluation

As unsupervised clustering methods with no predefined labels are notoriously hard to evaluate, multiple methods were applied for evaluation. First, silhouette analysis was performed, which measures cluster separation by determining the average distance of each element in a cluster from all other clusters. A silhouette close to 1 indicates excellent clustering, while a score closer to 0 indicates some overlap between clusters, and a negative score indicates that the data was clustered incorrectly. To find the k-value, or number of clusters for which the clusters are most separated from each other, this analysis was performed for k-values from two to ten using tslearn^18^ functions based on Rousseeuw^22^. After determining the best k-value with the highest silhouette score and performing the clustering, the similarity of the resulting clusters was analyzed further using Davies-Bouldin Index^23^. This index is built from the separation and compactness of the clusters. Low values of the index indicate a homogeneous structure and good separation between the clusters.

#### Statistical analysis

The Shapiro-Wilk test was used to test the normal distribution of the force and duration values obtained from the chair rise dataset. Since the dataset contained non-normally distributed data, the null hypothesis was rejected and the non-parametric Mann-Whitney test was used to compare the clustered groups. A p-value of < 0.01 was defined as statistically significant. This analysis was performed in Python using the SciPy package.

## Results

### Study population

A total of 251 participants were enrolled at baseline (t0). Four participants dropped out after 6 months (t1), 19 participants dropped out after 24 months (t2), and another 37 participants did not participate in the 5CRT at t2. In addition, nine participants were excluded from data analysis due to errors in the recorded data. Ten participants were excluded because they did not complete the required five chair rises. This left 172 participants for analysis with a mean age of $76.9 \pm 3.4$ years. The participants were 73 males (42%) with mean Hand Grip Strength (HGS) of $33.88 \pm 7.1$ kg and 97 females (58%) with HGS of $18.25 \pm 4.31$ kg. Statistics data about the study population is shown in Table 2.

**Table 2:** Statistic data about the study population, including minimum (Min), maximum (Max), mean (Mean), and standard deviation (SD) of age in years, weight in kilograms (kg), height in meters (m), and 5CRT duration in seconds (s) as measured by the stopwatch.

	Min	Max	Mean	SD
Age [years]	71	89	76.9	3.4
Weight [kg]	48.1	126	75.1	13.4
Height [m]	1.45	1.91	1.67	0.09
5CRT stopwatch duration [s]	6.44	20.21	11.44	2.49

### K-means clustering

**Fig. 2:**
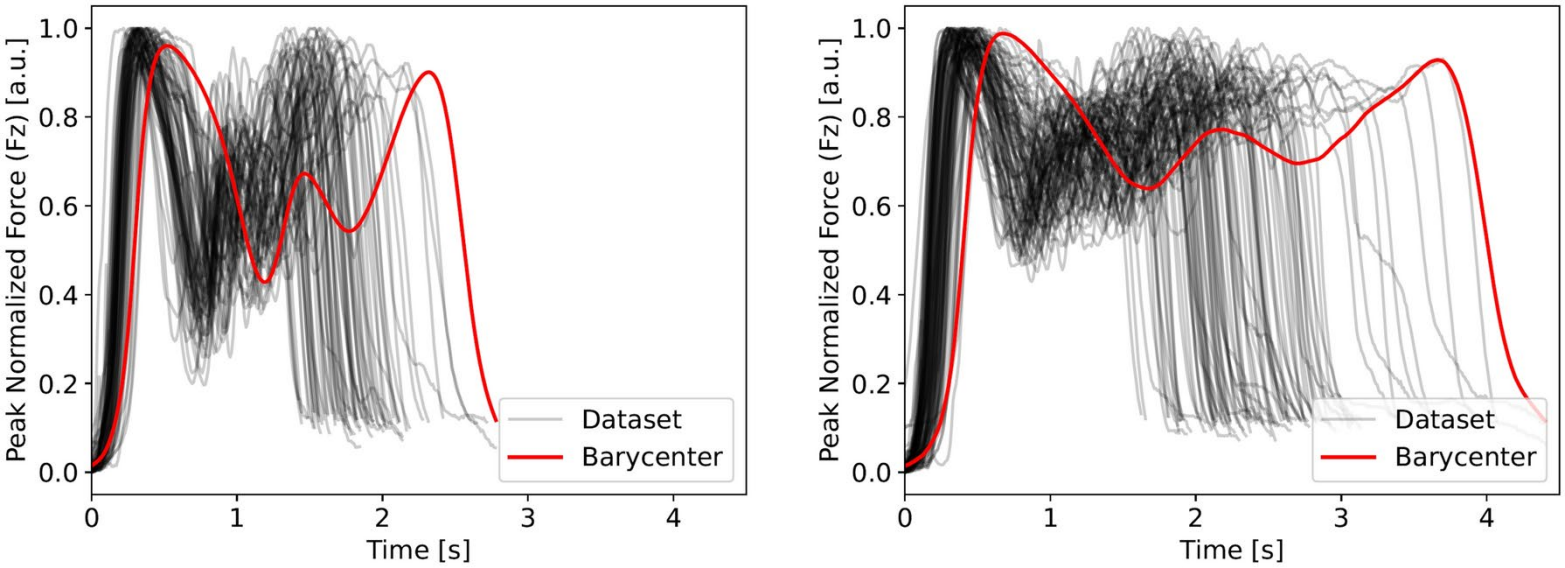
Barycenters and dataset of *Cluster 1* (left) and *Cluster 2* (right) after k-means clustering.

Using k-means clustering based on the DTW Barycenter Averaging (DBA) algorithm, a total of 860 chair rises were categorized into two clusters based on the applied force $F_z$ during the chair rise process. The participants were grouped based on a model trained on their first performed chair rise. The first performed chair rise was selected to highlight changes in the chair rise performance over time and enable evaluation of the model by clustering the remaining chair rises. To visualize the different clusters, Fig. 2 shows the soft-DTW Barycenters of both clusters based on the force $F_z$ of the first chair rise of each participant as recorded by the GRF plate.

Silhouette analysis of k-values ranging from two to ten showed that selecting two clusters was the best option, resulting in the definition of the two clusters found in this work. However, the silhouette score of 0.38 for $k=2$ shows that there is some overlap between clusters. To further evaluate the clustering, the Davies-Bouldin Index was determined to be 0.92, suggesting fairly well separated clusters with small overlaps.

**Fig. 3:**
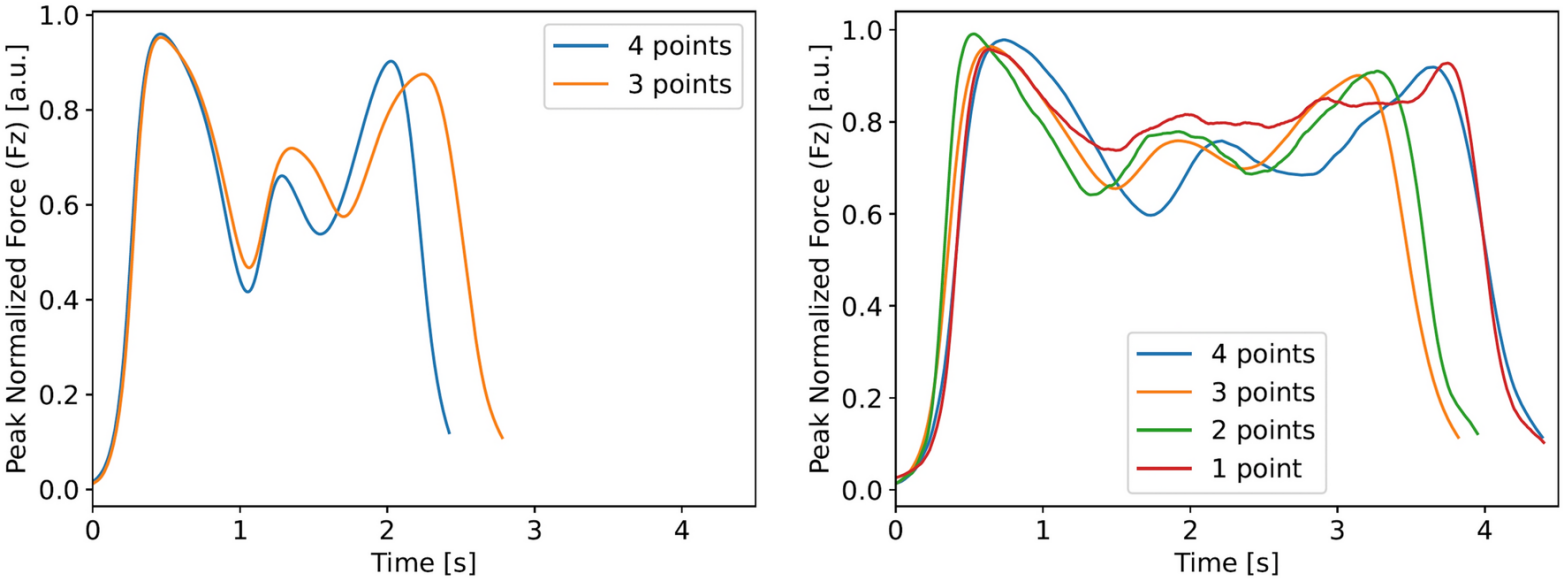
Barycenters of sub-datasets created by splitting the *Cluster 1* (left) and *Cluster 2* (right) datasets based on each scored category according to Guralnik et al.^4^ as shown in Table 1.

Ultimately, k-means clustering resulted in 72 participants being assigned to *Cluster 1* and 100 participants to *Cluster 2*. Further analysis shows 48% of the male participants were categorized in *Cluster 1* and 52% in *Cluster 2*. While only 37% of female participants where assigned to *Cluster 1* and 63% to *Cluster 2*. A significant difference (p$<0.01$) can be found in the 5CRT duration recorded by the stopwatch, where *Cluster 1* has a mean duration of $9.84 \pm 1.41$ s, while *Cluster 2* has a mean duration of $12.59 \pm 2.47$ s. To better understand the effect of 5CRT duration on the found clusters, the clusters are further divided by each score category as presented in Guralnik et al.^4^ and Table 1. Figure 3 shows the Barycenters for each of the resulting datasets. Note that *Cluster 1* does not contain any participants with a 5CRT duration $>13.69$ s, while *Cluster 2* contains all scored categories.

### Quantification of 5CRT clusters

**Fig. 4:**
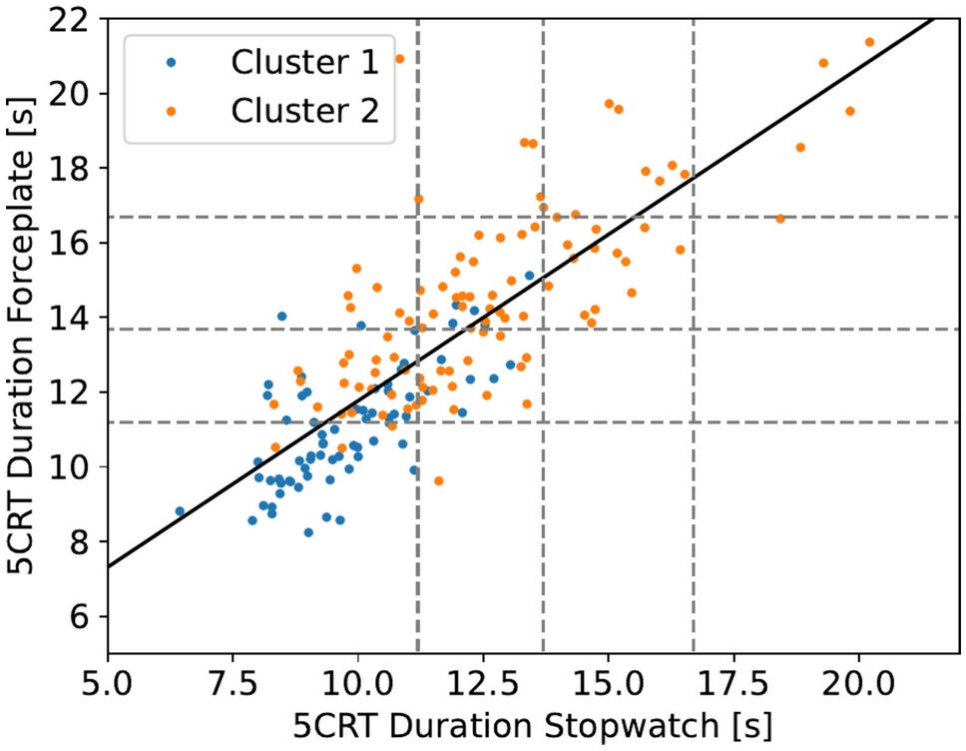
Comparison of 5CRT duration in seconds determined by stopwatch and by labeling of the GRF data, with the scoring time limits according to Guralnik et al.^4^ indicated as gray dashed lines.

Given the large sample size of 172 participants analyzed in this work, a comparison of the stopwatch recorded 5CRT duration with other labeled data is warranted, as suggested by Hellmers et al.^13^. Therefore, a comparison was made between the stopwatch recorded 5CRT duration and the duration estimated by labeling the GRF data. Both measurements are shown in Fig. 4 together with a linear fitted curve. In addition to comparing the two methods, this highlights the differences in how the stopwatch detects the clusters. The results of the linear regression can be found in the following equation:

$$\begin{aligned} t_{GRF} = 0.89\cdot t_{Stopwatch} + 2.86. \end{aligned}$$

**Table 3:** Mean and standard deviation of each participant’s normalized ground reaction force $F_z$ during all phases highlighted in Fig. 1, as well as p-value result of Mann-Whitney test.

	Cluster 1	Cluster 2	p-value
Mean force during “Seat-Unloading” [a.u.]	0.5 ± 0.18	0.55 ± 0.17	0.091
Mean force during “Seat-Off” [a.u.]	1.16 ± 0.13	1.15 ± 0.12	0.971
Mean force during “Stabilization” [a.u.]	0.39 ± 0.16	0.69 ± 0.13	* $2.45\cdot 10^{-21}$
Mean force during “Standing” [a.u.]	1.04 ± 0.09	1.01 ± 0.067	0.013

**Fig. 5:**
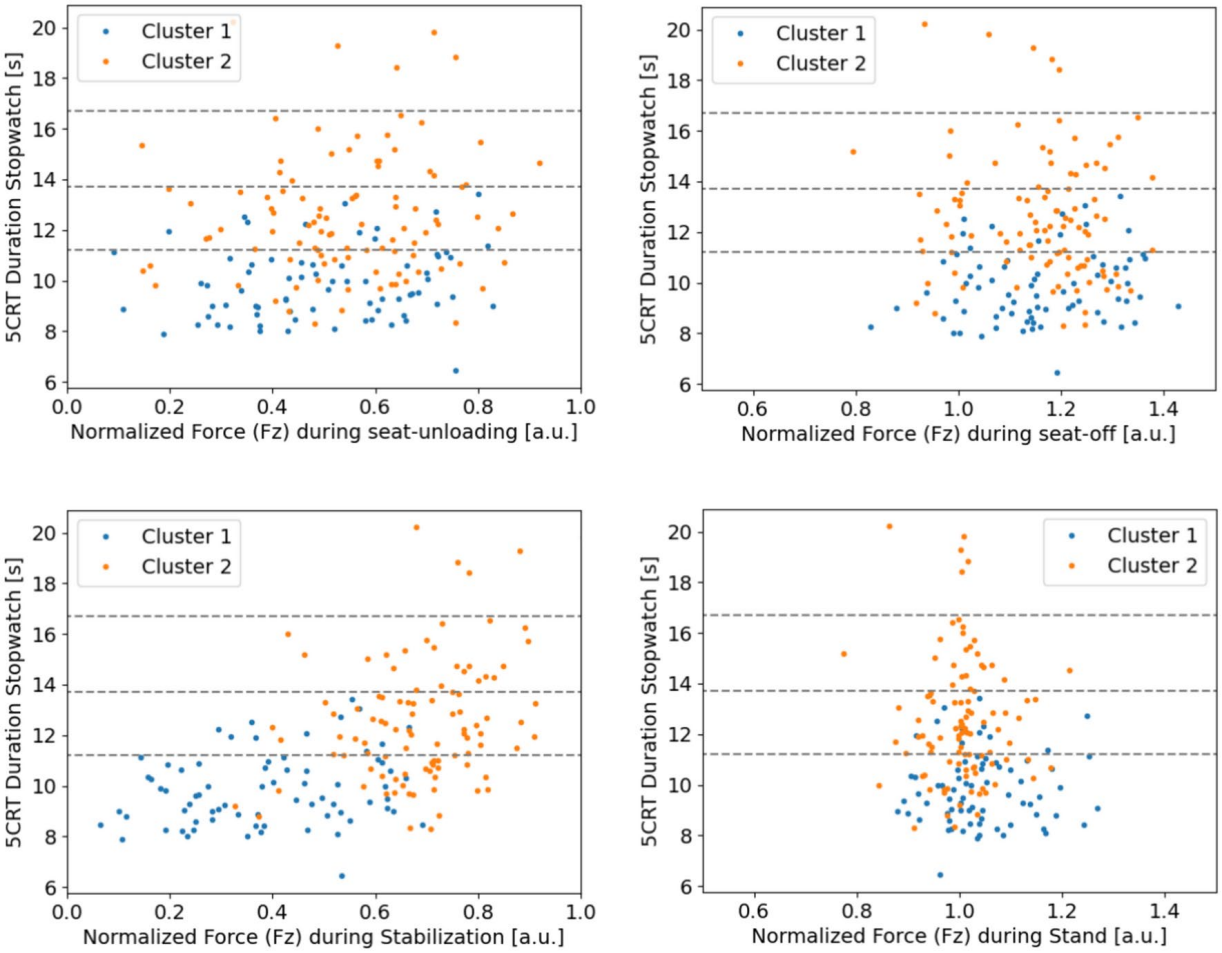
Normalized ground reaction force $F_z = F_{z,GRF}/m g$ during Seat-Unloading (top left), Seat-Off (top right), Stabilization (bottom left) and the Stand phase (bottom right) for all participants of each cluster, with the scoring time limits according to Guralnik et al.^4^ indicated as gray dashed lines.

To further quantify the differences in the clusters, a study of the force measured during different phases of the chair rise was made. For this, the peak ground reaction force $F_z$ during all phases of the chair rise previously identified in Fig. 1 were analyzed. Their mean value per cluster can be seen in Table 3. Additionally, the determined value of each participant is displayed in Fig. 5.

Finally, to evaluate the connection of the first chair rise to the overall performance of the participant, the remaining chair rises were clustered with the same model. The following Table 4 shows that the model was able to correctly assign $78\%$ of participants by their first chair rise. For these participants all chair rises were assigned the same cluster. These percentages are higher for *Cluster 2* ($81\%$) then *Cluster 1* ($75\%$). Only $6\%$ of all participants showed that the first chair rise does not belong to the cluster assigned to the rest. For *Cluster 1* this means $7\%$ of participants where assigned to *Cluster 1* because of their first chair rise, but all other chair rises where assigned to *Cluster 2*.

**Table 4:** Participants who have either all or fewer of the five chair rises assigned to the same cluster as their first chair rise.

	All	Four	Three	Two	Only first
*Cluster 1* (n = 72)	75%	10%	7%	1%	7%
*Cluster 2* (n = 100)	81%	5%	4%	5%	5%
*Total* (n = 172)	78%	7%	5%	3%	6%

## Discussion

Assessing the stability of a single performed chair rise can be difficult, if not impossible, for even experienced clinicians/researchers when observing the chair rise. The aim of this work was to investigate movement patterns in older adults during the 5CRT using k-means clustering and DTW. This method was applied to the vertical ground reaction force ($F_z$) measured by a force plate to investigate possible differences in movement strategies between participants and to identify potential stability or other age-related issues. The results revealed two distinct movement patterns, identified as *Cluster 1* and *Cluster 2*, that differed significantly in 5CRT duration and forces during the stabilization phases. These patterns have shown to be undetectable with conventional methods of assigning scores based on the 5CRT duration measured by a stopwatch and will be discussed in the following.

*Cluster 1* and *Cluster 2* have shown to be indistinguishable with conventional methods of assigning scores based on the 5CRT duration measured by a stopwatch, as proposed in Guralnik et al.^4^ and common in the SPPB. This fact can be seen by comparing the 5CRT duration measured by the stopwatch and that estimated by labeling the GRF data. While a linear relationship was observed between the two methods, an underestimation of the 5CRT duration by the stopwatch was found. It has been shown (in Fig. 4) that the GRF method is more accurate in grouping participants of *Cluster 1* into the best score category, while the stopwatch method increasingly groups participants of *Cluster 2* into better categories than the GRF method. This difference between the methods suggests that the human error introduced with the stopwatch method shifts the score by multiple seconds and can have a significant effect on the achieved score. While the scores defined by Guralnik et al.^4^, visible in Table 1, are based on the stopwatch method, this shift raises questions about the reproducibility of the stopwatch method. However, even when determining the 5CRT duration based on the GRF data, it could be seen, that the assigned clusters have a significant ($\text {p}<0.01$) difference in both duration measurements, but there is an overlap between the clusters—especially when using the 5CRT duration measured by the stopwatch. Therefore, different movement patterns are not solely a result of a longer 5CRT duration and additional analysis is needed to quantify the found clusters.

Statistical analysis of the force applied during the 5CRT proved that the vertical force $F_z$ applied during the seat-unloading, seat-off and stand phase are all not significant ($\text {p}>0.01$) to the cluster selection, while the vertical force applied in the first stabilization phase showed a significant difference between both clusters (p < 0.01, see Table 3). This implies that the clusters are defined by their difference in the force applied in the stabilization phase and the 5CRT duration as mentioned before. However, the effect of the 5CRT duration on the clustering is limited by the applied DTW metric. In summary, *Cluster 1* is characterized by both significantly ($\text {p}<0.01$) shorter 5CRT durations and lower vertical forces during stabilization. This movement pattern is therefore indicative of greater physical capacity, as participants in *Cluster 1* were able to transition efficiently between postures while maintaining stability. Suggesting that participants in *Cluster 1* are more capable of stabilizing their body weight during the stabilization phase and are therefore capable of completing the 5CRT faster then participants of *Cluster 2*. In contrast, *Cluster 2* participants showed significantly ($\text {p}<0.01$) longer 5CRT durations and higher vertical forces during the stabilization phase, indicating a challenge to stability control in this part of the 5CRT. This aligns with literature findings on dynamic balance control. Specifically, it took significantly longer for healthy older adults to acquire their stability in 5CRT^7^.

To build upon the previously discussed quantification of the clusters, it was investigated how indicative the first chair rise, on which the model was trained, is of the whole 5CRT of the participants (see Table 4). This was achieved by clustering the other chair rises (2–5) with the k-means model. It was found that participants assigned to *Cluster 2* by their first chair rise were more likely to be assigned to *Cluster 2* for all their chair rises. On the other hand, participants initial clustered within *Cluster 1* were more likely to have at least one of their chair rises in the 5CRT to be assigned to *Cluster 2*. This suggests that *Cluster 1* is more prone to change, suggesting that the movement pattern of *Cluster 1* is harder to maintain over the duration of the 5CRT.

Additional analysis shows that participants with the lowest values of physical performance found in the same study population by Diekmann et al.^14^ can be found within *Cluster 2* and show no changes in cluster assignment during the 5CRT. These participants showed the lowest level of performance in all tests (SPPB, TUG, 4mGS, and HGS). This indicates that the k-means clustering performed in this work could predict low physical performance and further analysis of the performance could prove that this method could replace or enhance several other physical performance tests.

As initially proposed in Hellmers et al.^13^ the DTW method proved to be worthwhile when investigating the movement patterns of the 5CRT and can improve upon previous clustering methods applying Euclidean distance measures.

## Limitations and future perspectives

A major influence on the performance of a chair rise is the height of the chair relative to the leg length of each participant. While the chair used to perform the 5CRT in our study was adjustable in height, thus reducing this effect, there could still be unknown variations in the height of the chair. Another factor that could affect the chair rises performed is the angle of the participants’ legs. Therefore, the predefined leg angle of $90^\circ$, which is the starting position for the first chair rise, may not represent the other chair rises. This starting position may be uncomfortable for some participants who perform subsequent chair rises from a more comfortable position. A similar result could be caused by a training effect, where participants improve their chair rise performance during the 5CRT, or based on their previously performed tests during the SPPB. The focus on the sit-to-stand part of the 5CRT to quantify the stabilization of each participant proved to be valuable. Quantification using the stand-to-sit could have been problematic as participants could ’fall’ back into the chair.

Estimating the duration of the 5CRT via the stopwatch also has some limitations, which could be the reasons for the offset found to the GRF measured 5CRT duration. One may be the announcement of the end of the test during the fifth and final chair rise when the stopwatch is used to measure the duration. While the stopwatch should stop after the last chair rise, there is reason to believe that sometimes the stopwatch was stopped prematurely before the participant was at rest. Additionally, the participants’ reaction time to the start signal could influence the stopwatch results. When considering the estimation of 5CRT duration using the labeled force plate data, the nature of the label identification could cause further discrepancies with the stopwatch. While the stopwatch may have stopped when the participant touched the chair, the labeled chair rise ended when the participant was at rest and no additional force was applied.

Contrary to the fixed limitations imposed by the nature of the performed 5CRT, the k-means model presented in this work can be further optimized. Given the nature of k-means clustering, the model could be improved by additional filtering and selection of the dataset used for training. Downsampling the dataset, or applying filters, such as a low-pass filter, could improve the computational time to train the model and improve the separation of clusters. Selecting participants of specific sex, height and other biological factors could also prove valuable. Most of the participants were healthy older adults, resulting in a possible underrepresentation of unhealthy participants in the construction of the k-means model. Therefore, the pre-selection of participants in the dataset used to train the model could further improve the ability of the model to detect unhealthy or infrequent movement patterns.

In future research, the quantification of the movement patterns by extended analysis of the different phases of the sit-to-stand and a more detailed study of the different clusters promise to improve the selection of the training data and thus the k-means model. Also, a study of the participants of each cluster within the time categories furthest from the cluster center - e.g. 4 and 3 points in *Cluster 2* could prove worthwhile. A detailed analysis of the COM displacement and other components derived from the recorded force plate data could also increase the knowledge about the two clusters found in this work, or highlight other patterns within these two. The inclusion of additional data channels such as inertial measurement units or surface electromyography may also be worthwhile to find more differences in the clusters. Additionally, it could be investigated how the clustering is affected by training the k-means algorithm on all five of the chair rises, or the variation between them. It may also be interesting to study the different phases of the sit-to-stand and stand-to-sit movements independently^24^.

To date, the use of HGS as a substitute for measuring lower limb muscle strength and physical function has been widely discussed in the field^25^. Therefore, given the HGS data collected in the evaluation, future work could also investigate a correlation between the clusters and participants’ HGS as an additional instrument to gain insight into the strategies used in 5CRT performed by older adults. However, given the large difference in HGS found between male and female participants in this work, this analysis might have to be performed for both groups individually, limiting the amount of data usable for the k-means.

Ultimately, the trained k-means model could assist untrained researchers or nurses in performing a detailed evaluation of 5CRT performance. Given the large number of participants in the assessment analyzed, additional models could be trained to evaluate the other tests performed during the SPPB. The use of DTW to detect multiple tests within a force plate data file combined with k-means models could support a detailed analysis of the SPPB test.

## Conclusion

By analyzing and comparing a total of 860 chair rises using Dynamic Time Warping and k-means clustering, these tools are very promising for assessing the 5CRT performance of older adults. The existence of different movement patterns among the participants was demonstrated using the k-means method based on the Dynamic Time Warping Barycenter Averaging algorithm. The method applied in this work can distinguish between two distinct patterns, which could provide further insight into the chair rise process in addition to the scoring method proposed in Guralnik et al.^4^. Therefore, additional analysis of the force applied during the chair rise test may improve the predictability of participants’ stability challenges based solely on the chair rise test. Although this increases the complexity of the assessment by requiring the force plate and additional data analysis, this data analysis could be performed, or supported by machine learning models such as the one created in this work. Therefore, the use of machine learning and the force plate during the 5CRT could ultimately reduce the number of performance tests required per participant to gather important information about their stability and health.

## Data Availability

The data supporting the results of this study are available from the corresponding author upon reasonable request.
